# A comparison of the performance of molecularly imprinted polymer nanoparticles for small molecule targets and antibodies in the ELISA format

**DOI:** 10.1038/srep37638

**Published:** 2016-11-24

**Authors:** Katarzyna Smolinska-Kempisty, Antonio Guerreiro, Francesco Canfarotta, César Cáceres, Michael J. Whitcombe, Sergey Piletsky

**Affiliations:** 1 Department of Chemistry, College of Science and Engineering, University of Leicester, LE1 7RH, UK

## Abstract

Here we show that molecularly imprinted polymer nanoparticles, prepared in aqueous media by solid phase synthesis with immobilised L-thyroxine, glucosamine, fumonisin B2 or biotin as template, can demonstrate comparable or better performance to commercially produced antibodies in enzyme-linked competitive assays. Imprinted nanoparticles-based assays showed detection limits in the pM range and polymer-coated microplates are stable to storage at room temperature for at least 1 month. No response to analyte was detected in control experiments with nanoparticles imprinted with an unrelated template (trypsin) but prepared with the same polymer composition. The ease of preparation, high affinity of solid-phase synthesised imprinted nanoparticles and the lack of requirement for cold chain logistics make them an attractive alternative to traditional antibodies for use in immunoassays.

It is now well over twenty years since the first demonstration that molecularly imprinted polymers (MIPs) can be used as the recognition material (essentially as a replacement for antibodies) in assays for clinically significant drugs (diazepam and theophylline)^1^. While this seminal paper clearly illustrated the principle, the assays described were unlikely to present a threat to established methods using antibodies. The MIPs were prepared as “bulk” monoliths, either by thermal or photochemical polymerisation and subjected to wasteful grinding and sieving to obtain irregularly-shaped particles < 25 μm in size. Competitive binding with ^3^H-labelled analogues in organic solvents was used to establish a “molecularly imprinted sorbent assay” (MIA), with quantification of the analytes, in the μM concentration range, by scintillation counting. Antibody-based assays (such as ELISA) however are performed under aqueous conditions without the need for radiolabelled tracers. The assays can have much lower detection limits than MIA and readout is typically achieved by optical density measurements in a plate reader. The Achilles heel of antibody-based assays may indeed be the antibodies themselves. They are expensive to produce and purify; raising antibodies against a new target may take months and involve animal experiments; and they require careful storage and handling or their binding ability can be readily lost. Recent advances in the synthesis of MIP nanoparticles (nanoMIPs) have overcome the perceived drawbacks of the ground bulk MIPs used by Vlatakis *et al*.^1^ Indeed the solid-phase approach, using immobilised templates^2^, has allowed the synthesis of nanoMIPs to be carried out equally successfully in both organic^3^ and aqueous media^4^. The nanoparticles are selected on the basis of their affinity for the template, which is re-useable, and the synthesis is suitable for scale-up and automation^3^,^4^. Being chemical entities, additional functional layers may be created during the synthesis to modify the properties of the particles without affecting their recognition ability^5^,^6^. This raises that question: how do nanoMIPs prepared for small molecule templates compare with commercial antibodies in enzyme-linked competitive assays?

## Results and Discussion

**Figure 1:**
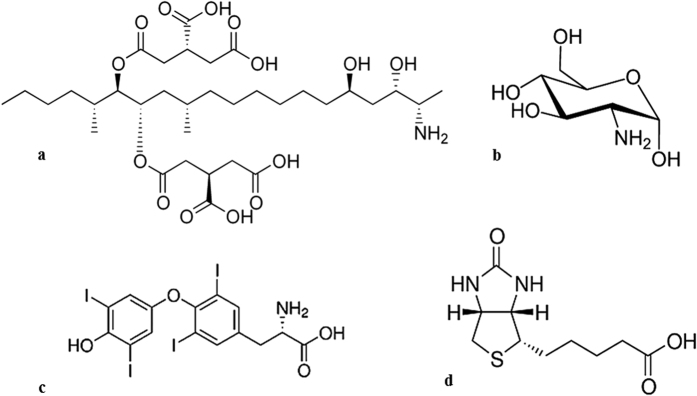
Structures of the analytes used as templates in this study: (**a**) fumonisin B2, (**b**) glucosamine, (**c**) l-thyroxine, (**d**) biotin.

In order to address the question posed above, we selected four chemically diverse target molecules for which commercial antibodies are available: fumonisin B2, glucosamine, l-thyroxine, and biotin (Fig. 1). Commercial sources were identified for monoclonal antibodies in the case of fumonisin B2 and l-thyroxine, while only polyclonal antibodies could be sourced in the case of glucosamine and biotin. For nanoMIP synthesis, templates were immobilised onto γ-aminopropyltrimethoxysilane-derivatised glass beads *via* their primary amino group with glutaraldehyde, followed by selective reduction with sodium cyanoborohydride, except in the case of biotin, which was coupled to the aminosilane-derivatised glass beads following activation of the carboxyl group with EDC/NHS. NanoMIP synthesis was conducted in aqueous media using the same monomer mixture (except for fumonisin B2, where acrylic acid was replaced with *N*-(3-aminopropyl)methacrylamide, see Methods section) initiated with persulfate/TEMED. As a control polymer, nanoMIPs were prepared using the same monomer composition against an unrelated template (trypsin) in order to obtain sufficient particles for use in comparative experiments (the presence of a template is necessary in order for particles to be isolated following the washing and elution steps^3^).

Solid-phase synthesis of nanoMIPs is a rapid method for the preparation of research quantities of antibody-like materials. Preparation of a batch of silylated glass beads takes 3–4 days while template immobilisation and the polymerisation and isolation of high affinity nanoMIPs can be achieved in 1–2 days. Since sufficient solid phase can be prepared in one batch to be divided amongst a number of templates, nanoMIPs for multiple targets can therefore be prepared within a two week period in contrast to the 3–6 months timescale for antibody production against each new target.

**Figure 2:**
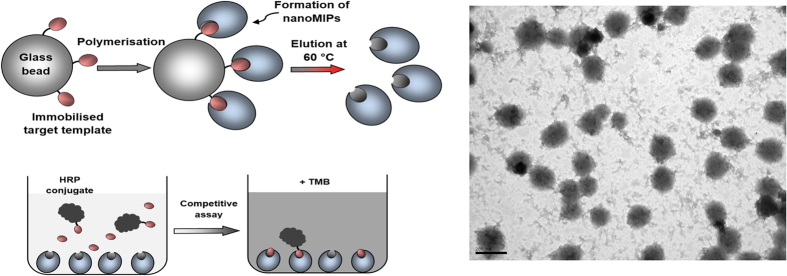
(Left) Schematic representation of nanoMIPs synthesis and competitive enzyme-linked assay; (Right) TEM image of nanoMIPs imprinted with l-thyroxine, scale bar = 200 nm.

In order to compare the performance of nanoMIPs with antibodies in a practically relevant scenario, we elected to see how well both classes of affinity materials perform in enzyme-linked competitive binding assays. To this end, conjugates of each target molecule with horseradish peroxidase [EC 1.11.1.7] (HRP) were prepared via EDC/NHS coupling, targeting the same functional group used in immobilisation to the solid-phase (–NH_2_ in the case of fumonisin B2, glucosamine and l-thyroxine and –CO_2_H in the case of biotin). Assays were conducted in 96-well microplates using 3,3′,5,5′-tetramethylbenzidine (TMB) as the substrate for HRP. Assays using nanoMIPs were based on the protocol previously developed for vancomycin using imprinted nanoparticles^7^. The synthesis of nanoMIPs and the assay protocol are shown schematically in Fig. 2, along with a representative TEM image.

There have been relatively few reports of the preparation of polymers imprinted with our chosen templates: fumonisin B2 MIPs for solid-phase extraction have been prepared by bulk polymerisation in acetonitrile^8^; glucosamine MIPs, prepared in aqueous buffer, have been reported for extraction of the monosaccharide from chicory root^9^; l-thyroxine-imprinted films have been prepared on electrodes for sensing applications^10^,^11^; while biotin MIPs have been prepared as sol-gel magnetic core-shell particles^12^, as thin films^13^,^14^ and in bulk format^15^. In contract to these diverse methods of synthesis, the imprinted polymer nanoparticles prepared in this work were all prepared in aqueous media, irrespective of the solubility or otherwise of the template molecules in water. This is possible because the formation of imprinted polymer occurs at the surface of the glass beads, removing the necessity for the template molecule to be soluble in the polymerisation medium. While the presence of water has long been thought to interfere with the formation of high affinity sites imprinted with small molecules, in the case of solid-phase synthesis, high affinity imprinted particles are formed in almost every case. The nanoparticles are isolated as an aqueous suspension which can be used as a direct replacement for antibodies. In a parallel study, the effect of the size (molar mass) of the templates used for imprinting in aqueous media was investigated^16^. It was found that nanoparticles prepared in this manner could be used in the biomimetic ELISA format to create effective assays for horseradish peroxidase (44 kDa), cytochrome C (12 kDa) and biotin (244.31 g mol^−1^) as well as vancomycin (1449.3 g mol^−1^)^7^, however for the smallest template studied (melamine, 126.12 g mol^−1^), aqueous-based imprinting was not effective. For this template, nanoMIPs prepared in organic media, grafted with a shell of PEG methacrylate, could be used to prepare a satisfactory assay for melamine with a detection limit of 25 nM.

**Figure 3:**
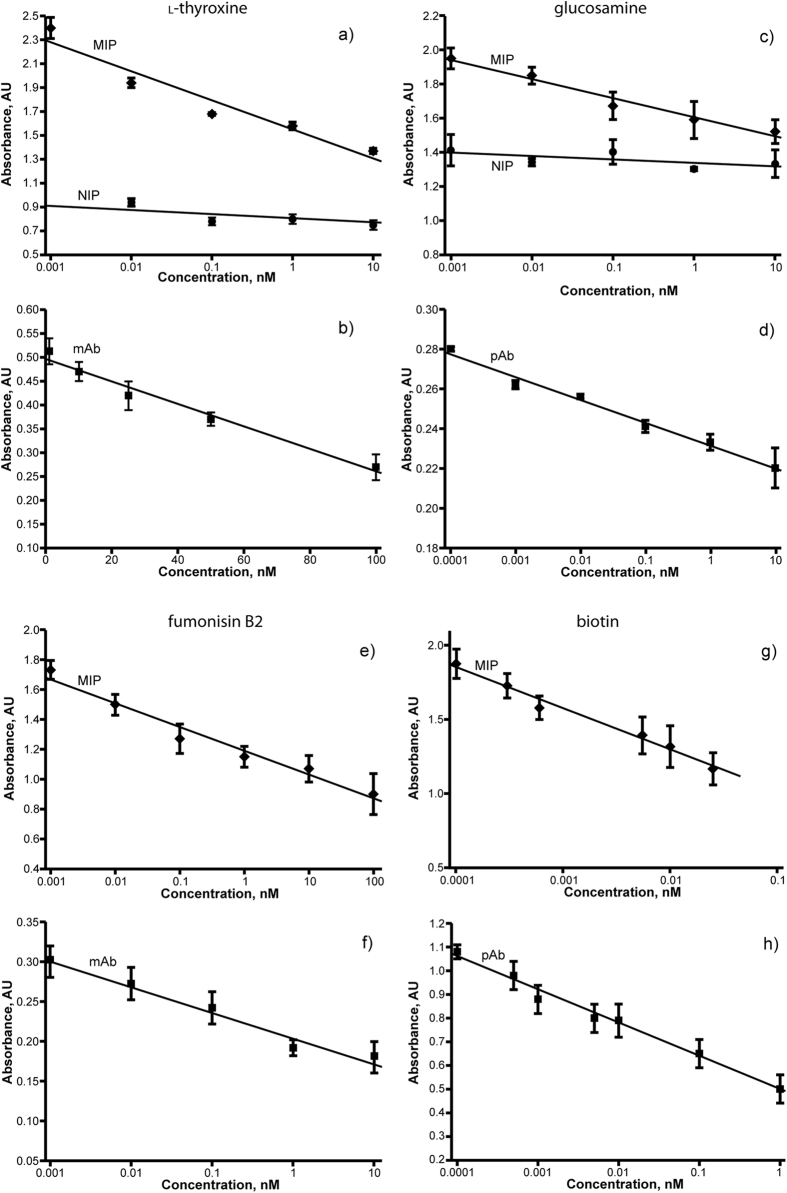
Calibration plots determined in enzyme-linked competitive assay formats for: l-thyroxine with (**a**) l-thyroxine-imprinted polymer nanoparticles (MIP) or trypsin-imprinted particles (NIP) or (**b**) monoclonal antibody (mAb) for l-thyroxine; glucosamine with (**c**) glucosamine-imprinted polymer nanoparticles (MIP) or trypsin-imprinted particles (NIP) or (**d**) polyclonal antibodies (pAb) for glucosamine; fumonisin B2 with (**e**) fumonisin B2-imprinted polymer nanoparticles (MIP) or (**f**) monoclonal antibodies (mAb) for fumonisin B2; biotin with (**g**) biotin-imprinted polymer nanoparticles (MIP) or (**h**) with polyclonal antibodies (pAb) for biotin. Error bars represent ±1 standard deviation and are for experiments performed in triplicate. Data for biotin-MIPs (g) is also included in a report where the effect of template size on aqueous solid-phase imprinting was investigated^16^, (see above).

For the comparative assays, the same HRP conjugates were used in experiments with both nanoMIPs and antibodies. Blocking and washing conditions were optimized in separate experiments and details of these are presented in Table 1. The variations in assay protocols for nanoMIPs and antibodies are not significant and differ mainly in the composition of blocking and washing buffers. All components of these solutions are commonly used in antibody-based assays. The optimized protocols were then used to perform competitive assays. Briefly, microplates coated with either nanoMIPs or antibodies were tested in an enzyme-linked assay using competition between the free target and the corresponding HRP-analyte conjugate. The solutions of analyte were added to the wells at the same time as the conjugate. The concentration of free analyte was typically varied over the range 1 pM to 100 nM. As shown in Fig. 3, the results clearly indicate that competition for binding of free analyte and its HRP conjugate was observed over a broad range of concentrations, and assay responses were found to be linearly proportional to the analyte concentration when plotted on a logarithmic concentration scale. As such, nanoMIPs are suitable for use in highly sensitive enzyme-linked assays for the target analytes.

Figure 3a shows the calibration curve for the enzyme-linked competitive assay for l-thyroxine with nanoMIPs. As was stated above, control experiments were performed with nanoMIPs prepared with the same monomer composition but imprinted against an unrelated template (trypsin)^17^. These particles are referred to as non-imprinted polymer (NIP) to distinguish them from the target-imprinted materials. The extent of binding of the l-thyroxine-HRP conjugate to the trypsin imprinted MIPs (NIP) is less than for binding to thyroxine-imprinted MIPs and, more importantly, is not affected by the presence of free L-thyroxine in solution. The lack of a specific response to L-thyroxine in control experiments is a good indication of the specificity of the assay. The nanoMIP-based assay showed linearity over the range 1 pM to 10 nM l-thyroxine, with a limit of detection (LoD) of 8 pM, calculated from the value of three times the standard deviation of the control (without free l-thyroxine). This result is similar to that previously seen for vancomycin^7^. Compared to monoclonal antibodies (Fig. 3b), the MIP-based assay was 3 orders of magnitude more sensitive. Unfortunately we could not find published data on ELISA protocols with this antibody to verify our findings. However, other thyroxin assays (from BIO-RAD)^18^ were used to measure this analyte at picomolar concentrations. It is possible that the reduced affinity of antibody-based assay in our experiments is linked to batch-to-batch variations or to denaturing of the antibody during transportation or storage. We can conclude however that nanoMIPs for l-thyroxin performed better than the antibodies tested in our laboratory and at least as well as the best examples of assays published earlier.

Figure 3c shows the calibration plot for the enzyme-linked competitive assay for glucosamine with nanoMIPs. As in the previous case, the binding of glucosamine-HRP conjugate to trypsin-imprinted nanoMIP (NIP) is lower than to glucosamine-imprinted MIPs and is not sensitive to the concentration of free glucosamine in solution. This lack of specific response to glucosamine in the control is a good indication of the specificity of the assay. In addition, no response was detected in the nanoMIPs-based assay when incubated with glucose or fructose (this is in contrast to the antibody-based assay which showed significant cross-reactivity with these analytes). The nanoMIPs-based assay showed linearity from 0.1 pM to 1 nM of glucosamine with a limit of detection of 0.4 pM, calculated from the value of three times the standard deviation of the control (without free glucosamine). The antibody-based assay showed a comparable sensitivity, with detection limit of 0.3 pM (Fig. 3d).

Figure 3e shows the calibration plot for the nanoMIP-based enzyme-linked competitive assay for fumonisin B2. The nanoMIPs-based assay showed linearity from 1 pM to 100 nM of fumonisin B2 with a limit of detection of 6 pM. No response to fumonisin was detected in the case of nanoMIPs imprinted with trypsin (data not shown). The antibody-based assay showed a four times higher detection limit - 25 pM (Fig. 3f).

Figure 3g shows the calibration plot for the nanoMIP-based enzyme-linked competitive assay for biotin. The assay based on nanoMIPs showed linearity from 0.1 pM to 30 pM with a limit of detection of 1.2 pM. No response to biotin was detected in the case of nanoMIPs imprinted with trypsin (data not shown). The antibody-based assay had slightly higher detection limit - 2.5 pM (Fig. 3h).

The MIP particle sizes and the limits of detection and working ranges of the nanoMIP and antibody-based assays are summarised in Table 2. As can be seen from these data, the performance of nanoMIPs was as good as, or better than antibodies in practically all cases. These tests clearly indicate that nanoMIPs synthesised by the solid-phase approach can be used as direct substitutes for monoclonal antibodies in ELISA.

Further experiments were performed in order to evaluate the stability of the MIP nanoparticles and the coatings prepared from them. Microplates with nanoMIPs imprinted against biotin were stored at room temperature for 1 month and tested as described above. The results showed that stored microplates could still be used in the biotin assay over the same concentration range with no deterioration in detection limit. The ability of nanoMIPs to work in assays after prolonged storage at room temperature could be beneficial for applications in tropical climates and/or in locations where access to freezers (cold chain delivery) is limited.

Small molecules (below ~10 kDa) are generally non-immunogenic, requiring conjugation to a carrier (protein or other biomacromolecule) in order to stimulate an immune response on immunisation of a host animal^19^. While *in vitro* methods of antibody selection are also capable of producing highly selective antibodies for small molecules^20^, this also relies on biological systems (such as phage display) in order to refine the selection process (SELEX) over several generations. In contrast, molecular imprinting is a method relying on synthetic chemistry and self-assembly. The tools for selection of functional monomers include an array of molecular modelling techniques that be used to greatly improve the likelihood of successful imprinting through a process of *in silico* design^21^. In addition, the formation of imprinted nanoparticles by solid phase synthesis is a rapid method that produces soluble particles bearing surface accessible binding sites and is capable of automation^2^,^3^,^4^. High affinity material is produced by virtue of an in-built affinity separation step, the template may be re-used, and the surface chemistry can be modified chemically without affecting the recognition properties of the MIPs^5^,^6^. The resultant particles are suitable for applications where antibodies are traditionally used, including sensors^22^,^23^,^24^,^25^ and assays^7^,^16^,^26^. Imprinted nanoparticles have also been shown to selectively detect mammalian cell types by their expression of antigens^27^,^28^.

The application of MIPs in diagnostic assays was recently reviewed^29^. A number of enzyme-linked assays for small molecules that used MIPs in place of antibodies were reported, however in all cases MIPs were prepared by *in situ* preparation of the imprinted polymer as a film within the microplate. This is clearly problematic in terms of ensuring reproducibility between wells and efficient template removal; moreover polymer formation is limited by the incompatibility of the microplates with monomers and solvents commonly used in imprinting. The solid-phase nanoMIPs, by contrast are easily immobilised in a reliable fashion by pipetting a known volume of their solution into each well and allowing the solution to dry^7^. For the film-based assays, the lowest LoD was reported for ractopamine (33 pM)^30^ with other reported LoDs lying between 0.76 nM (for tribenuron-methyl)^31^ to 1.2 μM for acrylamide^32^. The LoDs for the nanoMIP-based assays reported here (0.4–8.1 pM) therefore compare favourably with the most sensitive MIP-based ELISAs reported to date.

## Conclusions

This study demonstrates that molecularly imprinted nanoparticles (nanoMIPs) for low molar mass analytes may be prepared by solid-phase synthesis^2^,^3^ in aqueous media. The presence of water is normally considered to be unfavourable to the imprinting of hydrophilic templates. The formation of high-affinity imprinted nanoparticles, even in aqueous media appears to be a feature of the solid-phase imprinting approach. We have also shown that the nanoMIPs produced in this way can be used in the development of highly sensitive assays for low molecular weight targets such as biotin, fumonisin B2, glucosamine and l-thyroxine. The developed assays allowed the accurate determination of target analytes at picomolar concentrations. The results confirmed that nanoMIPs can be used as viable alternatives to antibodies in the ELISA format, showing similar to, or better performance than, the natural molecules. Furthermore, nanoMIP-based assays possess much higher stability, allowing their storage and transportation even in the absence of a cold chain. We believe these results serve as a strong endorsement for considering the industrial application of nanoMIPs in diagnostics platforms.

## Methods

### Synthesis of molecularly imprinted nanoparticles (nano-MIPs)

#### Preparation of template-derivatised glass beads

The glass beads were modified according to the protocol described previously^4^. In the steps described below, 0.4 mL solution was used per gram of glass beads. Briefly, first the glass beads were activated by boiling in 1 M NaOH for 15 min, washed with double-distilled water followed by acetone, and then dried. The beads were then incubated overnight in a solution of APTMS (2% v/v in dry toluene), washed with acetone, dried and subsequently incubated for 2 hours in a solution of GA in PBS (pH 7.4). The template was immobilized on the surface of glass beads by incubation of the beads in a solution of the appropriate template (1 mg mL^−1^) in PBS (pH 7.4) overnight at 4 °C. Afterwards, sodium cyanoborohydride was added to the solution of beads/template in PBS at 1 mg mL^−1^ and incubated for 30 min. Biotin was immobilized through EDC/NHS chemistry after the silanization step with APTMS. For this, 10 and 15 molar excess of EDC and NHS respectively were added to a 0.2 mg mL^−1^ solution of biotin in water and allowed to stand for 15 min prior to addition to the amine-derivatized glass beads. The pH of the biotin/glass beads solutions was adjusted to 7.4, and reaction was allowed to proceed for 2 hours. Finally template-modified glass beads were washed with double-distilled water, dried, and stored at 4 °C until use.

#### Synthesis of nanoMIPs

The polymerization mixture for the preparation of nanoMIPs comprised: NIPAM (39 mg), BIS (2 mg), TBAm (33 mg dissolved in 2 mL of ethanol), AA (2.23 μl for all templates except fumonisin B2) and NAPMA (2.2 mg only for fumonisin B2). The components were dissolved in water (100 mL), sonicated for 5 min, and degassed by bubbling with nitrogen for 30 min. Then 50 mL of this solution was added to 60 g of glass beads bearing the immobilized template. The polymerization was initiated by the addition of a solution (0.5 mL) of APS (60 mg/mL) containing TEMED (30 μL mL^−1^). The monomer mixture was allowed to polymerize at ambient temperature (20 °C) for 1.5 h. After this time, the beads were transferred into an SPE cartridge (60 ml) fitted with a 20 μm porosity PE frit. Unreacted monomers and other low affinity materials were removed by washing with double-distilled water (10 × 50 mL) at ambient temperature. Next, the temperature was raised to 60 °C and the fractions of high affinity nanoparticles were collected by washing with pre-warmed water at 60 °C (4 × 20 mL). The size of the nanoparticles was determined by dynamic light scattering (DLS) using a Zetasizer Nano (Nano-S) from Malvern Instruments Ltd (Malvern, UK).

### Immobilisation of nanoMIPs at the surface of microplate wells

Imprinted polymer nanoparticles (40 μL, 0.06 mg mL^−1^) were dispensed into the wells of a 96-well Nunclon microplate, and left to dry overnight at ambient temperature. Antibodies (50 μL, 0.005 mg mL^−1^) were dispensed into the polystyrene microplates and incubated for 3 hours.

### Competitive assay

The procedure for conducting enzyme-linked assays with nanoMIPs was carried out as described previously^3^. The procedure for antibodies was carried out in accordance with standard protocols^4^. The details of these procedures are summarized in Table 1. The absorbance (ABS) was measured for each well at a wavelength of 450 nm using UV-VIS microplate reader (Dynex, UK). All experiments were performed in triplicate. Nanoparticles imprinted against an unrelated template (trypsin) were used in control experiments.

Full experimental details, including optimisation of the antibody-based assays, can be found in the Supplementary information.

## Additional Information

**How to cite this article**: Smolinska-Kempisty, K. *et al*. A comparison of the performance of molecularly imprinted polymer nanoparticles for small molecule targets and antibodies in the ELISA format. *Sci. Rep.* **6**, 37638; doi: 10.1038/srep37638 (2016).

**Publisher’s note:** Springer Nature remains neutral with regard to jurisdictional claims in published maps and institutional affiliations.

## Supplementary Material

Supplementary Information

## Figures and Tables

**Table 1:** Procedures for enzyme-linked assay with antibodies and with nanoMIPs^7^.

Steps	Procedure for antibodies	Procedure for nanoMIPs
1. Immobilisation	3 hours (from CBB)	24 hours (from water)
2. Wash	Wash buffer: 0.05% Tween 20 in PBS (3 × 200 μL) pH 7.2	Wash buffer: PBS (2 × 250 μL) pH 7.4
3. Blocking of wells	2% BSA in wash buffer (300 μL, 1 hour)	0.1% BSA, 1% Tween 20 in PBS (300 μL, 1 hour)
4. Wash	No washing	PBS (3 × 250 μL)
5. Preparation of solutions of target + conjugate (TC)	Target dilution: from 10^−5^ to 100 nM Conjugate dilution: HRP-B 1:800; HRP-LT 1:400; HRP-G 1:200; HRP-F 1:200	
6. Addition of TC solution	(50 μL, 1 hour)	(100 μL, 1 hour)
7. Wash	2% BSA in wash buffer (6 × 200 μL)	0.1% BSA, 1% Tween 20 in PBS (3 × 300 μL)
8. Addition of TMB	50 μL (30 min)	100 μL (10 min)
9. Stop solution (sulfuric acid)	2 M (50 μL)	0.05 M (100 μL)

**Table 2:** NanoMIP particle sizes (as determined by dynamic light scattering, DLS), detection limits and assay linearity ranges for nanoMIP and antibody-based assays.

Analyte	NanoMIPs	NanoMIP-based assay		Antibody-based assay	
	Particle diameter (DLS), nm	Detection limit, pM	Linearity range, pM	Detection limit, pM	Linearity range, pM
Biotin	103.7 ± 5.9	1.2	0.1–30	2.5	0.1–10^3^
Fumonisin B2	93.6 ± 3.9	6.1	1–10^4^	25	1–10^3^
Glucosamine	137.6 ± 6.4	0.4	0.1–10^3^	0.3	0.1–10^4^
L-Thyroxine	164.2 ± 10.9	8.1	1–10^4^	17.5 × 10^3^	10^3^–10^5^

## References

[b1] Vlatakis G., Andersson L. I., Müller R. & Mosbach K. Drug assay using antibody mimics made by molecular imprinting. Nature 361, 645–647 (1993). 8437624 10.1038/361645a0

[b2] Canfarotta F., Poma A., Guerreiro A. & Piletsky S. Solid-phase synthesis of molecularly imprinted nanoparticles. Nat. Protocols 11, 443–455 (2016). 26866789 10.1038/nprot.2016.030

[b3] Poma A. . Solid-Phase Synthesis of Molecularly Imprinted Polymer Nanoparticles with a Reusable Template-“Plastic Antibodies”. Adv. Funct. Mater. 23, 2821–2827 (2013). 26869870 10.1002/adfm.201202397 PMC4746745

[b4] Poma A., Guerreiro A., Caygill S., Moczko E. & Piletsky S. Automatic reactor for solid-phase synthesis of molecularly imprinted polymeric nanoparticles (MIP NPs) in water. RSC Adv. 4, 4203–4206 (2014). 26722622 10.1039/C3RA46838K PMC4693954

[b5] Moczko E. . Surface-modified multifunctional MIP nanoparticles. Nanoscale 5, 3733–3741 (2013). 23503559 10.1039/c3nr00354j PMC4724934

[b6] Moczko E., Guerreiro A., Piletska E. & Piletsky S. PEG-Stabilized Core-Shell Surface-Imprinted Nanoparticles. Langmuir 29, 9891–9896 (2013). 23855734 10.1021/la401891f PMC4719183

[b7] Chianella I. . Direct Replacement of Antibodies with Molecularly Imprinted Polymer Nanoparticles in ELISA - Development of a Novel Assay for Vancomycin. Anal. Chem. 85, 8462–8468 (2013). 23947402 10.1021/ac402102j PMC4720989

[b8] De Smet D., Dubruel P., Van Peteghem C., Schacht E. & De Saeger S. Molecularly imprinted solid-phase extraction of fumonisin B analogues in bell pepper, rice and corn flakes. Food Addit. Contam., Part A 26, 874–884 (2009). 10.1080/02652030902788920 19680963

[b9] Henry N., Favetta P., Delépée R., Seigneuret J. M. & Agrofoglio L. A. Synthesis of a molecularly imprinted polymer to isolate glucosamine from plant extracts by an ionic - non-covalent dual approach. Int. J. Cosmet. Sci. 37, 196–206 (2015). 25400098 10.1111/ics.12182

[b10] Prasad B. B., Tiwari M. P., Madhuri R. & Sharma P. S. Enantioselective quantitative separation of d- and l-thyroxine by molecularly imprinted micro-solid phase extraction silver fiber coupled with complementary molecularly imprinted polymer-sensor. J. Chromatogr. A 1217, 4255–4266 (2010). 20483419 10.1016/j.chroma.2010.04.055

[b11] Prasad B. B., Madhuri R., Tiwari M. P. & Sharma P. S. Layer-by-layer assembled molecularly imprinted polymer modified silver electrode for enantioselective detection of d- and l-thyroxine. Anal. Chim. Acta 681, 16–26 (2010). 21035598 10.1016/j.aca.2010.09.027

[b12] Uzuriaga-Sánchez R. J. . Magnetically separable polymer (Mag-MIP) for selective analysis of biotin in food samples. Food Chem. 190, 460–467 (2016). 26212997 10.1016/j.foodchem.2015.05.129

[b13] Elmlund L., Suriyanarayanan S., Wiklander J. G., Aastrup T. & Nicholls I. A. Biotin selective polymer nano-films. J. Nanobiotechnol. 12, Article No. 8 (2014). 10.1186/1477-3155-12-8 PMC3994413 24655809

[b14] Suriyanarayanan S., Petrone L., Ederth T. & Nicholls I. A. Biotinyl moiety-selective polymer films with highly ordered macropores. Chem. Commun. 49, 5274–5276 (2013). 10.1039/c3cc42235f 23633011

[b15] Wiklander J., Karlsson B. C. G., Aastrup T. & Nicholls I. A. Towards a synthetic avidin mimic. Anal. Bioanal. Chem. 400, 1397–1404 (2011). 21442363 10.1007/s00216-011-4907-5

[b16] Cáceres C. . Does size matter? Study of performance of pseudo-ELISAs based on molecularly imprinted polymer nanoparticles prepared for analytes of different sizes. Analyst 141, 1405–1412 (2016). 26796951 10.1039/c5an02018b

[b17] Guerreiro A. . Influence of Surface-Imprinted Nanoparticles on Trypsin Activity. Adv. Healthcare Mater. 3, 1426–1429 (2014). 10.1002/adhm.201300634 PMC4681057 24652761

[b18] Boelen A. . Measuring free thyroxine levels in neonatal heel-prick samples. Clin. Chim. Acta. 423, 51–55 (2013). 23588061 10.1016/j.cca.2013.04.004

[b19] Clementi M. E., Marini S., Condo S. G. & Giardina B. Antibodies against small molecules. Ann. Ist. Super. Sanità 27, 139–144 (1991). 1958021

[b20] Bradbury A. R. M., Sidhu S., Dubel S. & McCafferty J. Beyond natural antibodies: the power of *in vitro* display technologies. Nat. Biotech. 29, 245–254 (2011). 10.1038/nbt.1791 PMC3057417 21390033

[b21] Cowen T., Karim K. & Piletsky S. Computational approaches in the design of synthetic receptors - A review. Anal. Chim. Acta 936, 62–74 (2016). 27566340 10.1016/j.aca.2016.07.027

[b22] Basozabal I., Guerreiro A., Gomez-Caballero A., Aranzazu Goicolea M. & Barrio R. J. Direct potentiometric quantification of histamine using solid-phase imprinted nanoparticles as recognition elements. Biosens. Bioelectron. 58, 138–144 (2014). 24632140 10.1016/j.bios.2014.02.054

[b23] Korposh S. . Selective vancomycin detection using optical fibre long period gratings functionalised with molecularly imprinted polymer nanoparticles. Analyst 139, 2229–2236 (2014). 24634909 10.1039/c3an02126b PMC4684099

[b24] Altintas Z. . Detection of Waterborne Viruses Using High Affinity Molecularly Imprinted Polymers. Anal. Chem. 87, 6801–6807 (2015). 26008649 10.1021/acs.analchem.5b00989

[b25] Garcia-Mutio, . Molecularly Imprinted High Affinity Nanoparticles for 4-Ethylphenol Sensing. Procedia Eng. 120, 1132–1136 (2015).

[b26] Shutov R. V. . Introducing MINA - The Molecularly Imprinted Nanoparticle Assay. Small 10, 1086–1089 (2014). 24504940 10.1002/smll.201301996 PMC4719181

[b27] Kunath S. . Cell and Tissue Imaging with Molecularly Imprinted Polymers as Plastic Antibody Mimics. Adv. Healthcare Mater. 4, 1322–1326 (2015). 10.1002/adhm.201500145 25880918

[b28] Yin D. Y. . Surface-enhanced Raman scattering imaging of cancer cells and tissues via sialic acid-imprinted nanotags. Chem. Commun. 51, 17696–17699 (2015). 10.1039/c5cc05174f 26489719

[b29] Bedwell T. S. & Whitcombe M. J. Analytical applications of MIPs in diagnostic assays: future perspectives. Anal. Bioanal. Chem. 408, 1735–1751 (2016). 26590560 10.1007/s00216-015-9137-9 PMC4759221

[b30] Fang G. Z. . Substitution of Antibody with Molecularly Imprinted Film in Enzyme-Linked Immunosorbent Assay for Determination of Trace Ractopamine in Urine and Pork Samples. Food Anal. Meth. 4, 590–597 (2011).

[b31] Liu H. L., Ren L., Fang G. Z., Li H. Y. & Wang S. An enzyme-linked immunosorbent assay for the determination of tribenuron-methyl in water and soil using a molecularly imprinted film as an artificial antibody. Anal. Methods 5, 5677–5683 (2013).

[b32] Sun Q., Xu L. H., Ma Y., Qiao X. G. & Xu Z. X. Study on a biomimetic enzyme-linked immunosorbent assay method for rapid determination of trace acrylamide in French fries and cracker samples. J. Sci. Food Agric. 94, 102–108 (2014). 23633401 10.1002/jsfa.6204

