# Supplementary material for: A comparison of the performance of molecularly imprinted polymer nanoparticles for small molecule targets and antibodies in the ELISA format
Source: Sci Rep. 2016 Nov 24;6:37638. doi: 10.1038/srep37638 (PMC5121619; doi:10.1038/srep37638)
Supplement: Supplementary Information [file srep37638-s1.pdf]

A comparison of the performance of molecularly imprinted polymer nanoparticles for small molecule targets and antibodies in the ELISA format

Katarzyna Smolinska-Kempisty, Antonio Guerreiro, Francesco Canfarotta, César Cáceres,[a] Michael J. Whitcombe*, Sergey Piletsky*

*mw319@le.ac.uk; sp523@le.ac.uk

Department of Chemistry, College of Science and Engineering, University of Leicester, LE1 7RH, UK

[a]Current address: Department of Analytical and Inorganic Chemistry, Faculty of Chemical Sciences, University of Concepcion, Chile

Supplementary Information

Materials

Antibodies: anti-biotin pAb (goat) were from Sigma-Aldrich, UK; anti-fumonisin B2 mAb (mouse, monoclonal) was from INSIGHT Biotechnology Ltd UK, anti-D-glucosamine pAb (rabbit, polyclonal) was from Abcam, UK and anti-thyroxine mAb (mouse, monoclonal) from Abd Serotech, UK. Templates: D-glucosamine hydrochloride 99% (G), L-thyroxine 98%, fumonisin B2 were from Santa Cruz Biotechnology, USA, biotin was from Sigma-Aldrich, UK. Other materials: Acrylic acid (AA), *N*-isopropylacrylamide (NIPAm) *N*,*N*′-methylene-bis-acrylamide (BIS), *N*-tert-butylacrylamide (TBAm), horseradish peroxidase (HRP), ammonium persulfate (APS), tetramethylethylenediamine (TEMED), 3-aminopropyltrimethyloxysilane (APTMS), D-(-) fructose, D-(+) glucose 97%, sodium cyanoborohydride, sodium hydroxide (NaOH), glutaraldehyde (GA), bovine serum albumin (BSA), 3,3′,5,5′-tetramethylbenzidine used in the form of TMB liquid substrate system for ELISA (Sigma, UK, catalogue number T0440), Tween-20, (2-[morpholino]ethanesulfonic acid) (MES), *N*-hydroxysuccinimide (NHS), 1-ethyl-3-(3-dimethylaminopropyl)carbodiimide hydrochloride (EDC), *N*,*N*-

dimethylformamide (DMF) and acetone were from Sigma-Aldrich, UK. *N*-(3-aminopropyl)methacrylamide hydrochloride >98% (NAPMA) was from Polyscience Inc. Glass beads (Spheriglass A-Glass 2429 CP-00, 50-106 μm diameter) were from Blagden, UK. Phosphate buffered saline (PBS), consisted of phosphate buffer (0.01 M), potassium chloride (0.0027 M) and sodium chloride (0.14 M), pH 7.4 (Gibco Life technologies Ltd, UK) and carbonate-bicarbonate buffer (CBB), pH 9.5 (Sigma-Aldrich, UK) was prepared from buffer tablets. Type II purified water (Purelab Option, Veolia, UK) was used for the experiments. All chemicals and solvents were used without further purification. Microplates used for MIP-based assays were Nunclon 96 microwell plates (Thermo Scientific, UK) and for natural antibodies were polystyrene 96 microwell plates (MICR-TPF, ELKAY).

Synthesis of molecularly imprinted nanoparticles (nano-MIPs)

Preparation of template-derivatised glass beads

The glass beads were modified according to the protocol described previously.[1] In the steps described below, 0.4 mL solution was used per gram of glass beads. Briefly, first the glass beads were activated by boiling in 1M NaOH for 15 min, washed with double-distilled water followed by acetone, and then dried. The beads were then incubated overnight in a solution of APTMS (2% v/v in dry toluene), washed with acetone, dried and subsequently incubated for 2 hours in a solution of GA in PBS (pH 7.4). The template was immobilized on the surface of glass beads by incubation of the beads in a solution of the appropriate template (1 mg mL^{-1}) in PBS (pH 7.4) overnight at 4 °C. Afterwards, sodium cyanoborohydride was added to the solution of beads/template in PBS at 1 mg mL^{-1} and incubated for 30 min. Biotin was immobilized through EDC/NHS chemistry after the silanization step with APTMS. For this, 10 and 15 molar excess of EDC and NHS respectively were added to a 0.2 mg mL^{-1} solution of

biotin in water and allowed to stand for 15 min prior to addition to the amine-derivatized glass beads. The pH of the biotin/glass beads solutions was adjusted to 7.4, and reaction was allowed to proceed for 2 hours. Finally template-modified glass beads were washed with double-distilled water, dried, and stored at 4 °C until use.

Synthesis of nanoMIPs

The polymerization mixture for the preparation of nanoMIPs comprised: NIPAM (39 mg), BIS (2 mg), TBAm (33 mg dissolved in 2 mL of ethanol), AA (2.23 µl for all templates except fumonisin B2) and NAPMA (2.2 mg only for fumonisin B2). The components were dissolved in water (100 mL), sonicated for 5 min, and degassed by bubbling with nitrogen for 30 min. Then 50 mL of this solution was added to 60 g of glass beads bearing the immobilized template. The polymerization was initiated by the addition of a solution (0.5 mL) of APS (60 mg/mL) containing TEMED (30 µL mL^{-1}). The monomer mixture was allowed to polymerize at ambient temperature (20 °C) for 1.5 h. After this time, the beads were transferred into an SPE cartridge (60 ml) fitted with a 20 µm porosity PE frit. Unreacted monomers and other low affinity materials were removed by washing with double-distilled water (10 × 50 mL) at ambient temperature. Next, the temperature was raised to 60 °C and the fractions of high affinity nanoparticles were collected by washing with pre-warmed water at 60 °C (4 × 20 mL). The size of the nanoparticles was determined by dynamic light scattering (DLS) using a Zetasizer Nano (Nano-S) from Malvern Instruments Ltd (Malvern, UK).

Preparation of HRP-analyte conjugates

Four different conjugates (HRP-glucosamine (HRP-G), HRP-Fumonisin B2 (HRP-F), HRP-L-thyroxine (HRP-L-T), and HRP-biotin (HRP-B) were prepared according to the previously

described protocol.[2] HRP (1 mg) was dissolved in 0.1 M MES buffer, pH 6.0 (1 mL), then EDC (0.4 mg) and NHS (1.1 mg) were added. The reaction was allowed to proceed at ambient temperature for 15 min. Then the buffer was removed by ultrafiltration on a Millipore Amicon Ultra centrifugal filter unit (30 kDa MWCO). Next, the appropriate analyte (molar ratio of glucosamine, fumonisin B2 or L-thyroxine to HRP, 100:1) was dissolved in 10 mL PBS buffer at pH 7.4 and added to the activated HRP. The reaction was allowed to proceed for 2 hours at 4 °C. In the case of biotin, a stock solution of Biotin (0.2 mg mL^{-1}) was prepared in 0.1 M MES buffer, pH 6 (1 mL), to which (17.7 μL) of EDC (10 mg mL^{-1} in water) was added, followed by NHS (1.72 mg). The reaction was allowed to proceed at room temperature for 15 min. After the 15 min the mixture was added to a solution of 20 mL of HRP (0.6 mg/mL) in PBS buffer at pH 7.4 for 2 h. After coupling, all conjugates were washed (10 × 5mL PBS) on a Millipore Amicon Ultra centrifugal filter unit (30 kDa MWCO) to remove any free analyte. After washing, the conjugates were dissolved in deionized water (2 mL) and stored frozen at –18 °C until use.

Immobilisation of nanoMIPs at the surface of microplate wells

Imprinted polymer nanoparticles (40 µL, 0.06 mg mL^{-1} in water) were dispensed into the wells of a 96-well Nunclon microplate, and left to dry overnight at ambient temperature. Antibodies (50 µL, 0.005 mg mL^{-1} in CBB) were dispensed into the polystyrene microplates and incubated for 3 hours.

Competitive assay

The procedure for conducting enzyme-linked assays with nanoMIPs was carried out described previously.[2] The procedure for antibodies was carried out in accordance with

standard protocols.[3] The details of these procedures are summarized in Table 1 (see manuscript). The absorbance (ABS) was measured for each well at a wavelength of 450 nm using UV-VIS microplate reader (Dynex, UK). All experiments were performed in triplicate. Nanoparticles imprinted against an unrelated template (trypsin) were used in control experiments.

Stability of nanoMIP-coated microplates

To assess the stability of microplate wells coated with nanoMIPs, several microplates were prepared as described above with nanoMIPs imprinted against biotin and subjected to storage trials for 1 month at room temperature. This was followed by testing in competitive assay for biotin as described above.

References

1. Moczko, E., Poma, A., Guerreiro, A., Perez de Vargas Sansalvador, I., Caygill, S., Canfarotta, F., Whitcombe, M. J. & Piletsky, S., Surface-modified multifunctional MIP nanoparticles. *Nanoscale* **5**, 3733-3741 (2013).
2. Chianella, I., Guerreiro, A., Moczko, E., Caygill, J. S., Piletska, E. V., De Vargas Sansalvador, I. M. P., Whitcombe, M. J. & Piletsky, S. A., Direct Replacement of Antibodies with Molecularly Imprinted Polymer Nanoparticles in ELISA - Development of a Novel Assay for Vancomycin. *Anal. Chem.* **85**, 8462-8468 (2013).

ELISA based on antibodies - optimisation of methods

MATERIALS AND METHODS

Materials. Antibodies: anti-biotin (goat) and anti-hemoglobin (rabbit) were from Sigma, UK, anti-fumonisin (2A2, mouse IgG) was from INSIGHT Santa Cruz Biotechnology Ltd, anti-glucosamine Rb pAb was from Abcam UK and anti-thyroxine (mouse) from BIO-RAD.

Phosphate buffered saline (PBS, consisted of phosphate buffer (0.01 M), potassium chloride (0.00268 M), and sodium chloride (0.140 M), pH 7.4 (Gibco Life technologies Ltd, UK) and carbonate-biscarbonate buffer (CBB), pH 9.5 (Sigma-Aldrich, UK) was prepared from buffer tablets. Microplates used for antibodies were polystyrene 96microwell plates (MICR-TPF, ELKAY).

Competitive Assay

The procedure for antibodies was carried out on the basis of “ELISA technical guide and protocols” (*ELISA technical guide and protocols,* Thermo Scientific, www.thermo.com/pierce.). These procedures are presented in Table 1. The absorbance, ABS, (450nm) of each well was measured using a UV-vis microplate reader (Dynex, UK). Errors bars represent 1 standard deviation and were for all experiments obtained from repetition three to six times.

ELISA Assay

1. Prepare antibodies solution 0.005 mg/mL in CBB and PBS
2. Dispense 50µL antibodies solution into the polystyrene microplates for 3h
3. Wash the microplate two times with 250µL per well wash buffer. Wash buffer: 0.05% Tween20 in PBS, pH 7.2.

4. Add 300µL blocking buffer per well, cover the microplate and incubate 1h at room temperature. Blocking buffer: 2% BSA in wash buffer.

5. Prepare various concentrations free template solutions in PBS (0.0001-100nM). Add the same amount of conjugate to each of them (biotin and glucosamine conjugate dilution 1:200, L-thyroxine 1:400).

6. Remove the blocking buffer and wash the microplate three times with 300µL per well wash buffer.

7. Add 100µL per well template solutions. Cover the microplate and incubate in the dark 1h at room temperature.

8. Remove template solutions and wash the microplates three times with 300µL per well blocking solution.

9. Add 100µL per well TMB solution and incubate the microplate at room temperature until you can see blue color.

10. Stop the reaction by adding 100µL per well stop solution. Stop solution: 2M sulfuric acid.

11. Measure the absorbance of each well at 450nm.

ELISA assay with anti-biotin antibodies

Two different procedures for anti-biotin antibodies were tested and compared: typical procedure for antibodies (I) and this one, developed for nanoMIP (II), Table 1 in the publication.

Concentration for biotin conjugate, HRP-B was chosen, (Table S1, Figure S1). In the case of antibody procedure dilution 1 to 200 was used.

Table S1. Optimization of the concentration HRP-B in the blank assay. I: according to the procedure for the antibodies, II: according to the procedure for the MIPs

	$ABS_{antibodies}$ / $ABS_{WITHOUT\ antibodies}$			
Dilution	1 to 200	1 to 400	1 to 800	1 to 1600
I	11.52	4.88	3.12	1.64
II	6.03	6.61	6.00	5.65

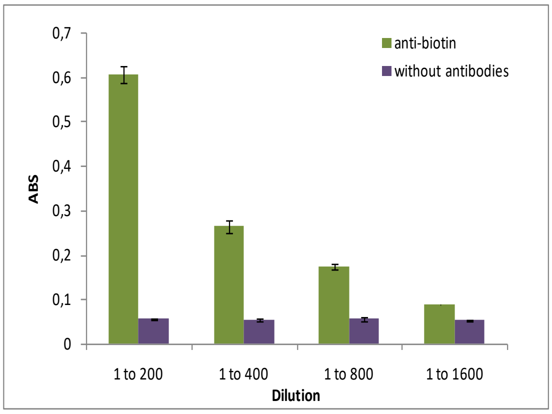

I

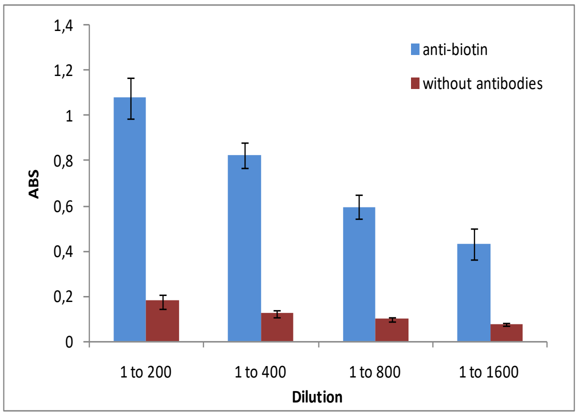

II

Figure S1. Optimisation of the concentration HRP-B in the blank assay. I: according to the procedure for the antibodies. II: according to the procedure for the MIPs.

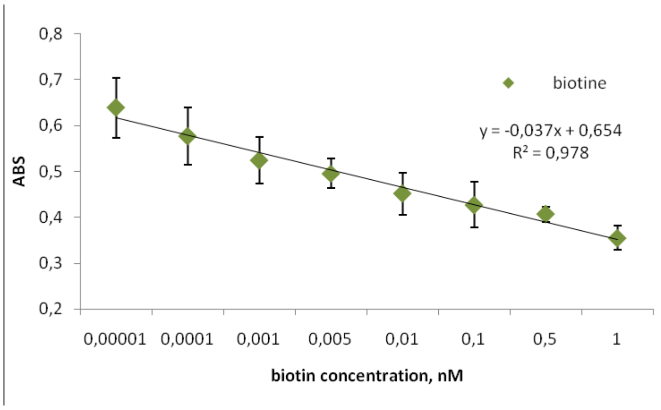

Figure S2. Calibration curves of the enzyme-linked anti-biotin antibodies based competitive assay performed with biotin, according to the procedure for the **antibodies**

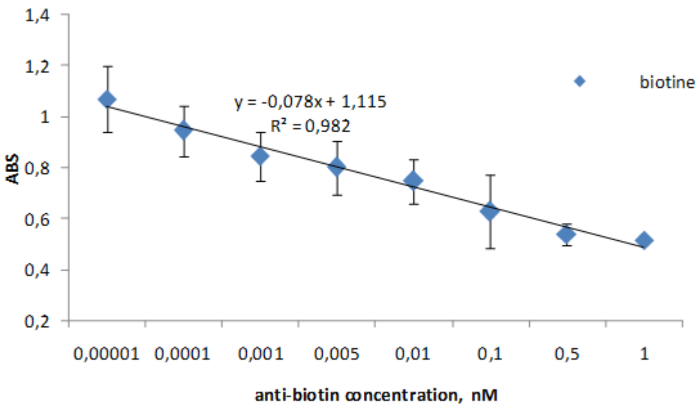

Figure S3. Calibration curves of the enzyme-linked anti-biotin antibodies based competitive assay performed with biotin. According to the procedure for the **nanoMIPs**

ELISA assay with anti-thyroxine antibodies

The anti-thyroxine antibodies were coated from PBS and CBB.

Table S2 and Figure S4 (A-B) show optimization of the L-T-conjugate dilution in the blank assay for antibodies. For the ELISA assay selected dilution 1 to 200. In the ELISA assay better results

obtained for antibodies coated from CBB (Figure S5). The linearity range observed from 1 to 100 nM.

Table S2. Absorbance factor in the blank assay for the dilution HRP-L-T. A: antibodies coated from PBS, B: antibodies coated from CBB

	Absorbance ratio $ABS_{antibodies} / ABS_{WITHOUT\ antibodies}$			
Dilution	1 to 200	1 to 400	1 to 800	1 to 1600
A	1.28	1.35	1.09	1.14
B	1.22	1.34	1.34	1.28

A B

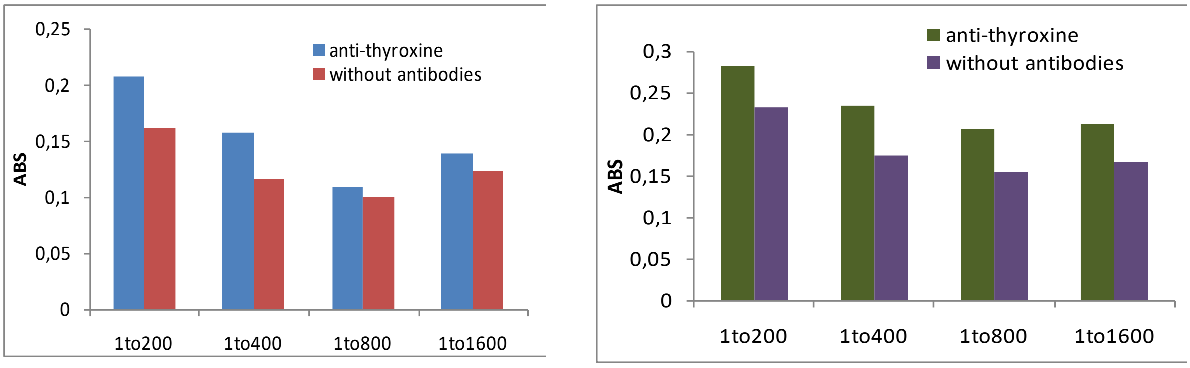

Figure S4. Optimisation of the dilution HRP-L-T in the blank assay. A: antibodies coated from PBS. B: antibodies coated from CBB

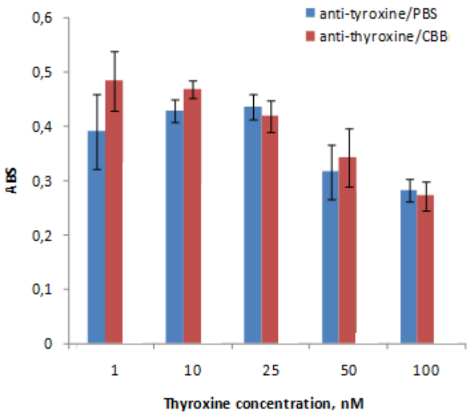

Figure S5. Enzyme-linked anti-thyroxine antibodies based competitive assay performed with L-thyroxine, antibodies coated from PBS and CBB

ELISA assay with anti-glucosamine antibodies

In the case of assay for glucosamine for antibodies for the best recognized conjugate dilution of 1 to 200 (Table S3. Figure S6 AB). For the ELISA assay antibodies were coated from CBB solution.

Table S3. Absorbance factory in the blank assay for the concentration HRP-G. A: antibodies coated from PBS. B: antibodies coated from CBB

	Absorbance ratio $ABS_{antibodies}$ / $ABS_{WITHOUT\ antibodies}$			
Dilution	1 to 200	1 to 400	1 to 800	1 to 1600
A	2.84	2.05	1.53	1.42
B	2.98	2.31	1.56	1.56

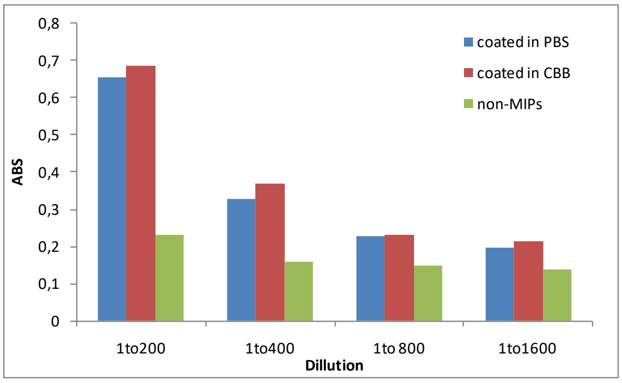

Figure S6. Optimization of the concentration of HRP-G in the blank assay. A: antibodies coated from PBS (blue). B: from CBB (red)

ELISA assay with anti-fumonisin B antibodies

In the case of anti-fumonisin antibodies regardless of the concentration of the solution whose were coated the biggest difference between the absorbance of antibodies samples and samples without antibodies were obtained for conjugate dilution of 1 to 200.

Table S4. Absorbance factor in the blank assay for the dilution HRP-F. A: antibodies coated from solution about concentration 5µg/ml. A': antibodies coated from solution about concentration 50µg/ml. A'': antibodies coated from solution about concentration 2000 µg/ml

	Absorbance ratio $ABS_{antibodies}$ / $ABS_{WITHOUT\ antibodies}$		
Dilution	1 to 200	1 to 400	1 to 800
A	2.56	2.34	1.61
A'	2.31	1.93	1.38
A''	2.25	1.75	1.29

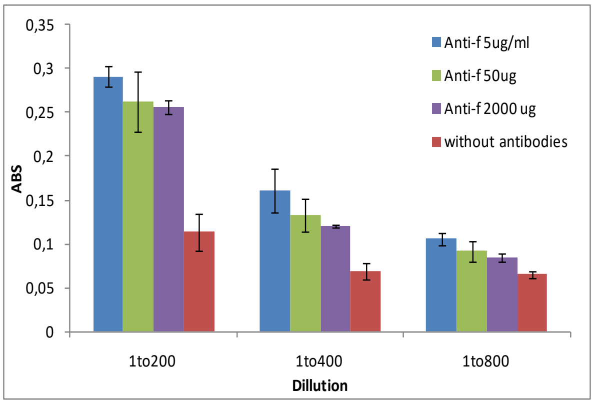

Figure S7. Optimisation of the dilution HRP-F in the blank assay, antibodies coated from CBB. A: solution concentration 5 µg/ml (blue), A’: 50 µg/ml (green), A’’: 2000 µg/ml (purple)

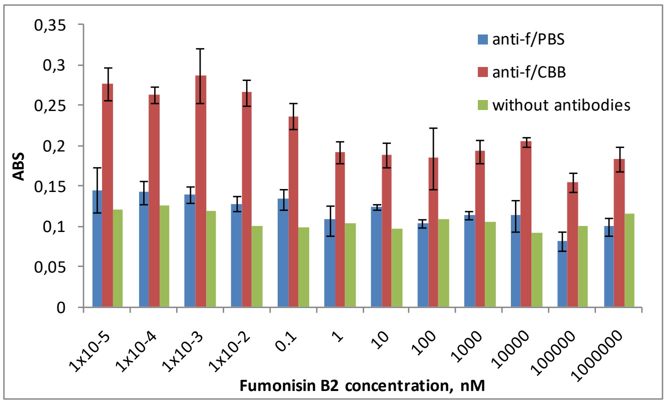

Figure S8. Enzyme-linked anti-fumonisin antibodies based competitive assay performed fumonisin B2, coated from PBS solution (blue), coated from CBB solution (red), without antibodies (green), all measure range

DLS measurements on nanoMIPs:

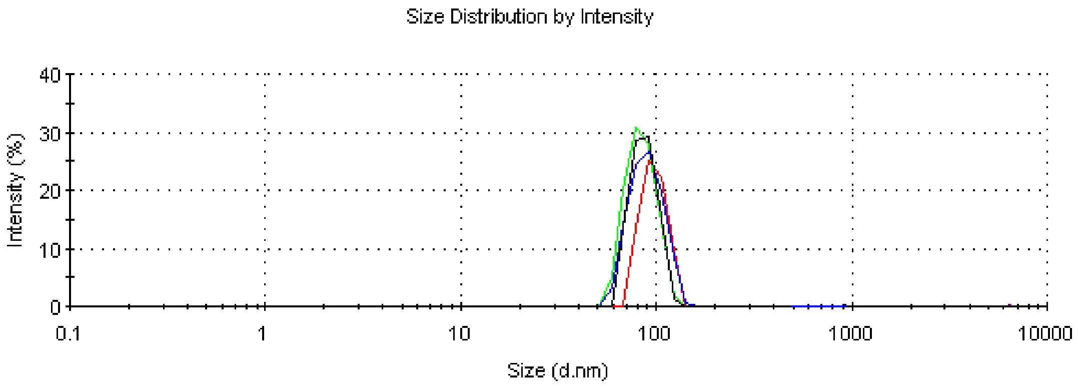

Figure S9. Data from DLS measurements carried out on the MIP nanoparticles imprinted with Fumonisin B2.

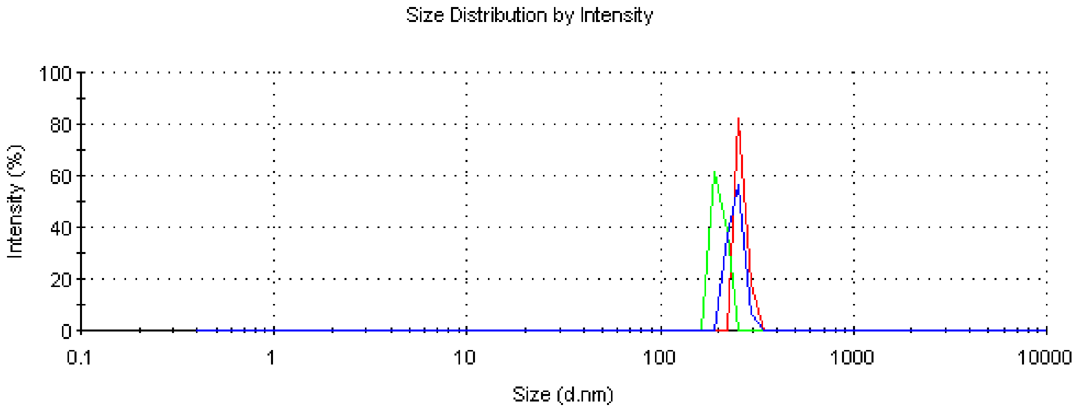

Figure S10. Data from DLS measurements carried out on the MIP nanoparticles imprinted with L-thyroxine.
